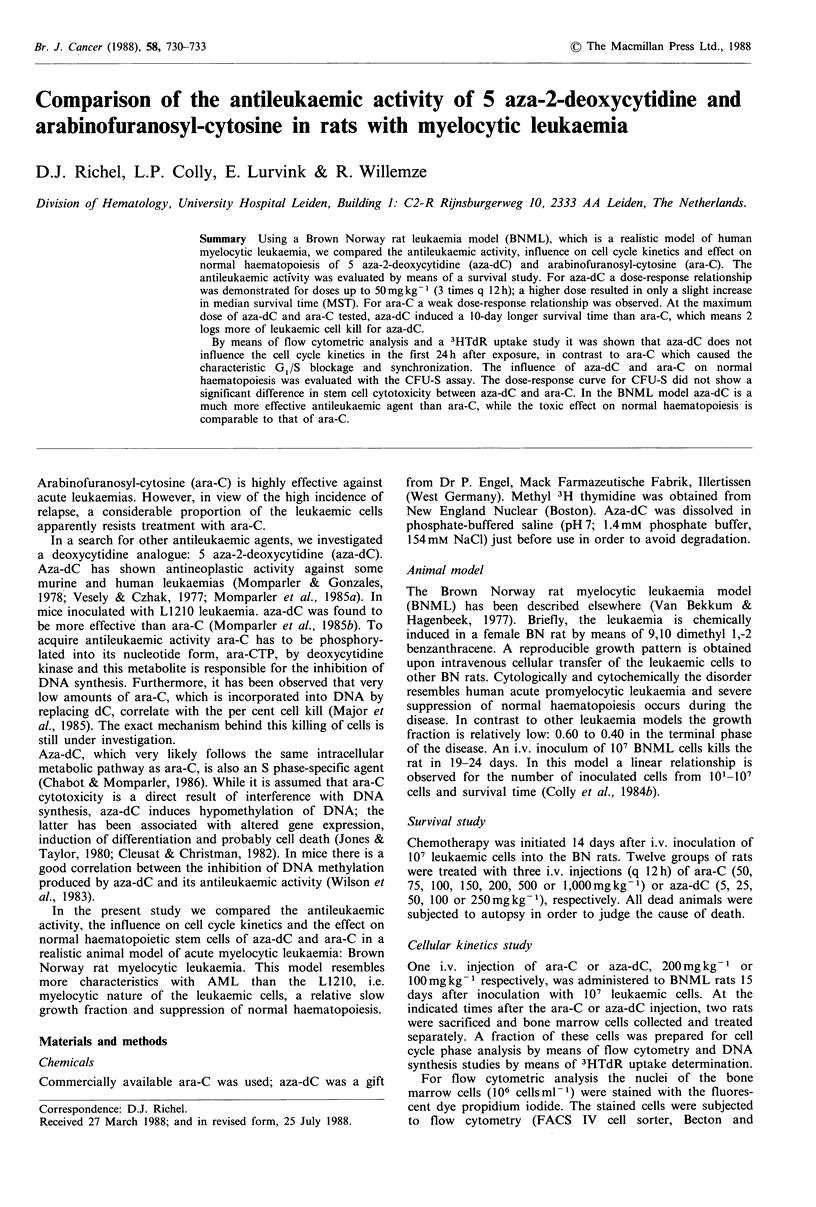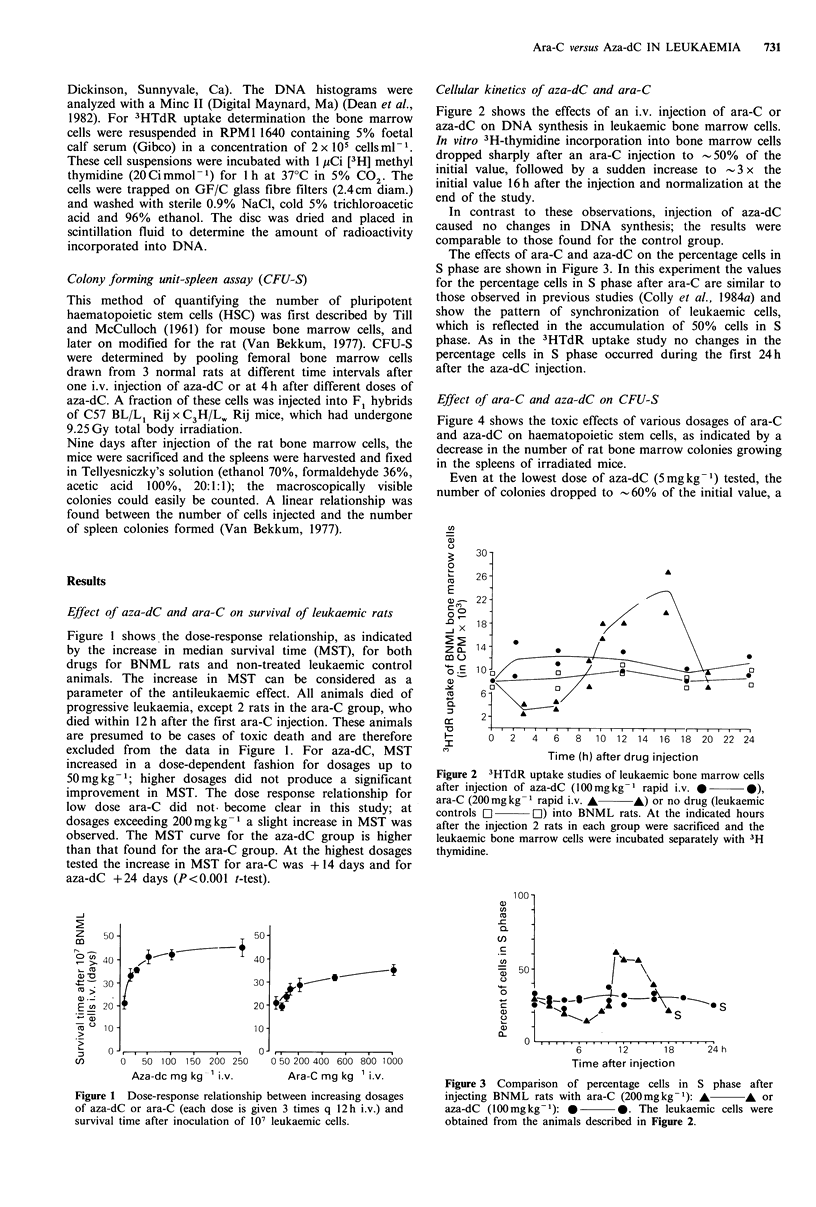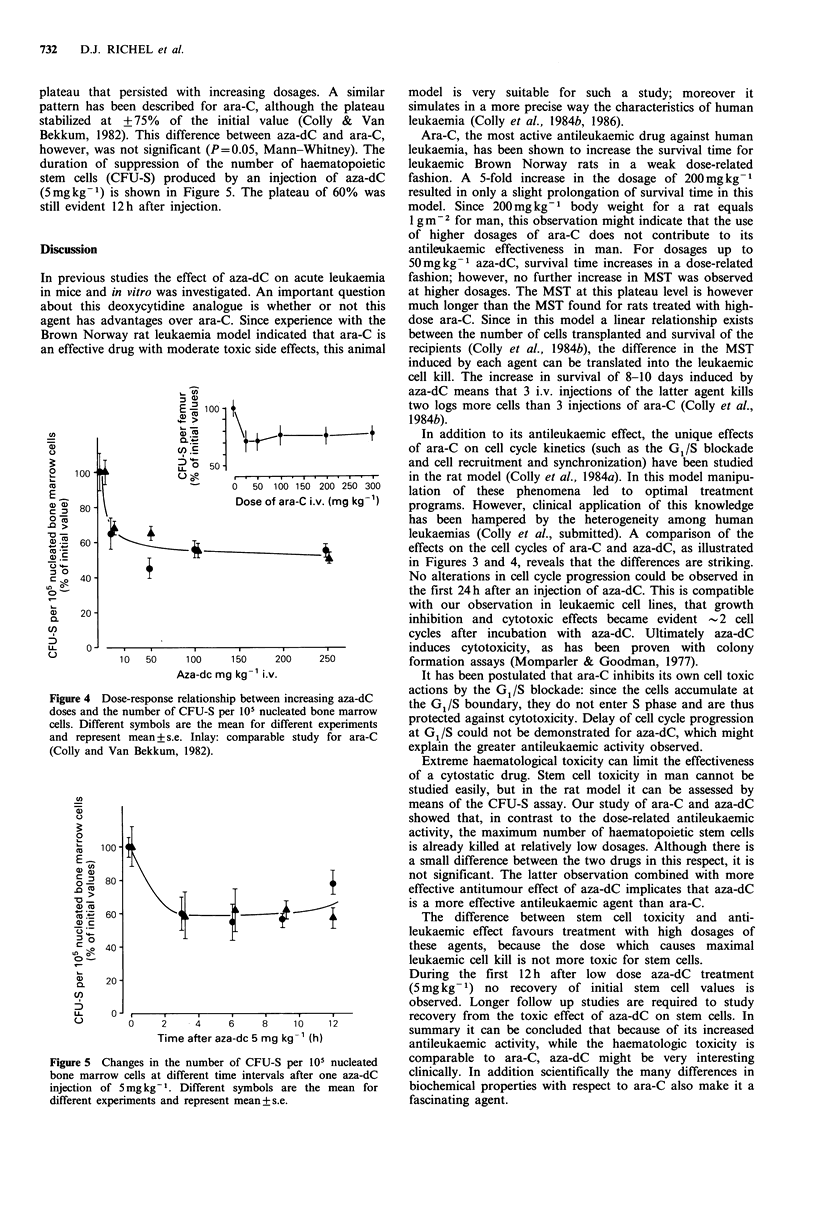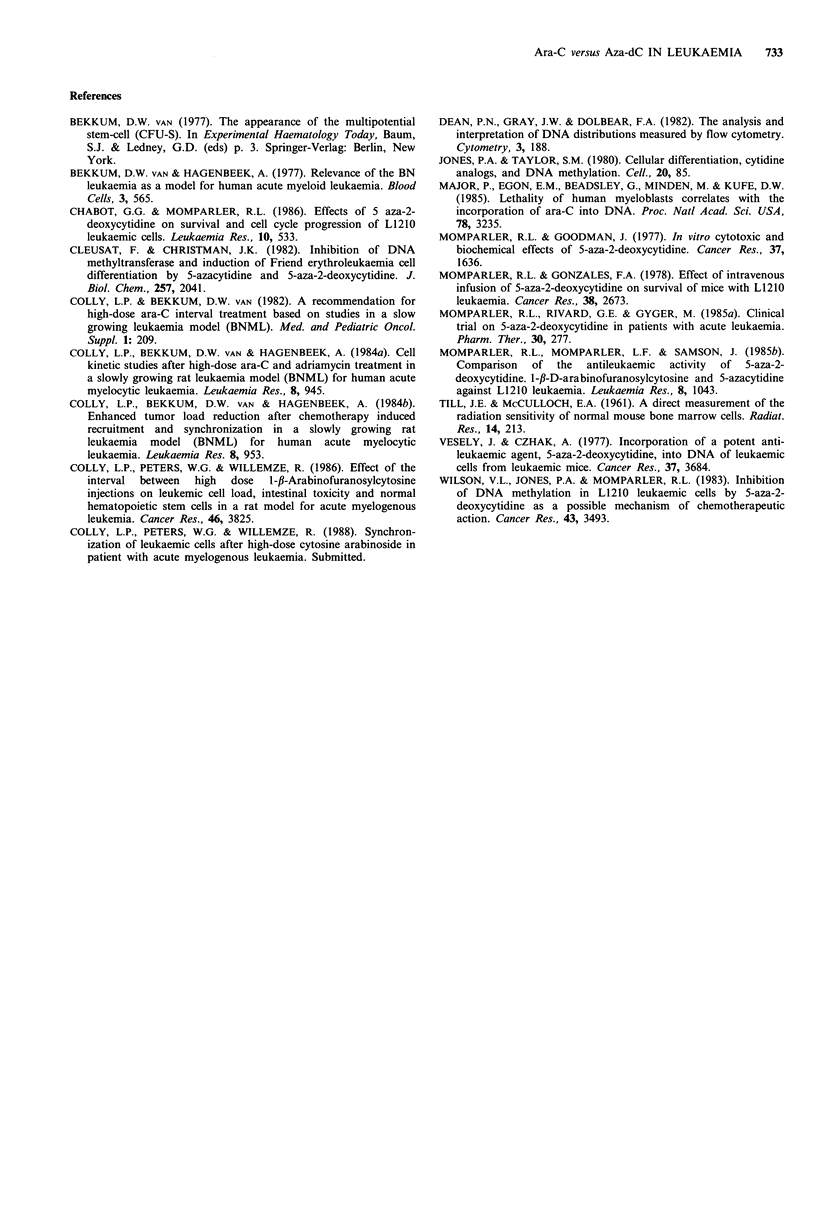# Comparison of the antileukaemic activity of 5 aza-2-deoxycytidine and arabinofuranosyl-cytosine in rats with myelocytic leukaemia.

**DOI:** 10.1038/bjc.1988.298

**Published:** 1988-12

**Authors:** D. J. Richel, L. P. Colly, E. Lurvink, R. Willemze

**Affiliations:** Division of Hematology, University Hospital Leiden, The Netherlands.

## Abstract

Using a Brown Norway rat leukaemia model (BNML), which is a realistic model of human myelocytic leukaemia, we compared the antileukaemic activity, influence on cell cycle kinetics and effect on normal haematopoiesis of 5 aza-2-deoxycytidine (aza-dC) and arabinofuranosyl-cytosine (ara-C). The antileukaemic activity was evaluated by means of a survival study. For aza-dC a dose-response relationship was demonstrated for doses up to 50 mg kg-1 (3 times q 12 h); a higher dose resulted in only a slight increase in median survival time (MST). For ara-C a weak dose-response relationship was observed. At the maximum dose of aza-dC and ara-C tested, aza-dC induced a 10-day longer survival time than ara-C, which means 2 logs more of leukaemic cell kill for aza-dC. By means of flow cytometric analysis and a 3HTdR uptake study it was shown that aza-dC does not influence the cell cycle kinetics in the first 24 h after exposure, in contrast to ara-C which caused the characteristic G1/S blockage and synchronization. The influence of aza-dC and ara-C on normal haematopoiesis was evaluated with the CFU-S assay. The dose-response curve for CFU-S did not show a significant difference in stem cell cytotoxicity between aza-dC and ara-C. In the BNML model aza-dC is a much more effective antileukaemic agent than ara-C, while the toxic effect on normal haematopoiesis is comparable to that of ara-C.


Br. J. Cancer (1988), 58, 730–733 © The Macmillan Press Ltd., 1988

### Comparison of the antileukaemic activity of 5 aza-2-deoxycytidine and arabinofuranosyl-cytosine in rats with myelocytic leukaemia

D.J. Richel, L.P. Colly, E. Lurvink & R. Willemze

*Division of Hematology, University Hospital Leiden, Building 1: C2-R Rijnsburgerweg 10, 2333 AA Leiden, The Netherlands.*

**Summary** Using a Brown Norway rat leukaemia model (BNML), which is a realistic model of human myelocytic leukaemia, we compared the antileukaemic activity, influence on cell cycle kinetics and effect on normal haematopoiesis of 5 aza-2-deoxycytidine (aza-dC) and arabinofuranosyl-cytosine (ara-C). The antileukaemic activity was evaluated by means of a survival study. For aza-dC a dose-response relationship was demonstrated for doses up to 50 mg kg$^{-1}$ (3 times q 12 h); a higher dose resulted in only a slight increase in median survival time (MST). For ara-C a weak dose-response relationship was observed. At the maximum dose of aza-dC and ara-C tested, aza-dC induced a 10-day longer survival time than ara-C, which means 2 logs more of leukaemic cell kill for aza-dC.

By means of flow cytometric analysis and a $^3$HTdR uptake study it was shown that aza-dC does not influence the cell cycle kinetics in the first 24 h after exposure, in contrast to ara-C which caused the characteristic $G_1$/S blockage and synchronization. The influence of aza-dC and ara-C on normal haematopoiesis was evaluated with the CFU-S assay. The dose-response curve for CFU-S did not show a significant difference in stem cell cytotoxicity between aza-dC and ara-C. In the BNML model aza-dC is a much more effective antileukaemic agent than ara-C, while the toxic effect on normal haematopoiesis is comparable to that of ara-C.

Arabinofuranosyl-cytosine (ara-C) is highly effective against acute leukaemias. However, in view of the high incidence of relapse, a considerable proportion of the leukaemic cells apparently resists treatment with ara-C.

In a search for other antileukaemic agents, we investigated a deoxycytidine analogue: 5 aza-2-deoxycytidine (aza-dC). Aza-dC has shown antineoplastic activity against some murine and human leukaemias (Momparler & Gonzales, 1978; Vesely & Czhak, 1977; Momparler *et al.*, 1985*a*). In mice inoculated with L1210 leukaemia. aza-dC was found to be more effective than ara-C (Momparler *et al.*, 1985*b*). To acquire antileukaemic activity ara-C has to be phosphorylated into its nucleotide form, ara-CTP, by deoxycytidine kinase and this metabolite is responsible for the inhibition of DNA synthesis. Furthermore, it has been observed that very low amounts of ara-C, which is incorporated into DNA by replacing dC, correlate with the per cent cell kill (Major *et al.*, 1985). The exact mechanism behind this killing of cells is still under investigation.

Aza-dC, which very likely follows the same intracellular metabolic pathway as ara-C, is also an S phase-specific agent (Chabot & Momparler, 1986). While it is assumed that ara-C cytotoxicity is a direct result of interference with DNA synthesis, aza-dC induces hypomethylation of DNA; the latter has been associated with altered gene expression, induction of differentiation and probably cell death (Jones & Taylor, 1980; Cleusat & Christman, 1982). In mice there is a good correlation between the inhibition of DNA methylation produced by aza-dC and its antileukaemic activity (Wilson *et al.*, 1983).

In the present study we compared the antileukaemic activity, the influence on cell cycle kinetics and the effect on normal haematopoietic stem cells of aza-dC and ara-C in a realistic animal model of acute myelocytic leukaemia: Brown Norway rat myelocytic leukaemia. This model resembles more characteristics with AML than the L1210, i.e. myelocytic nature of the leukaemic cells, a relative slow growth fraction and suppression of normal haematopoiesis.

### Materials and methods

### *Chemicals*

Commercially available ara-C was used; aza-dC was a gift from Dr P. Engel, Mack Farmazeutische Fabrik, Illertissen (West Germany). Methyl $^3$H thymidine was obtained from New England Nuclear (Boston). Aza-dC was dissolved in phosphate-buffered saline (pH 7; 1.4 mM phosphate buffer, 154 mM NaCl) just before use in order to avoid degradation.

### *Animal model*

The Brown Norway rat myelocytic leukaemia model (BNML) has been described elsewhere (Van Bekkum & Hagenbeek, 1977). Briefly, the leukaemia is chemically induced in a female BN rat by means of 9,10 dimethyl 1,-2 benzanthracene. A reproducible growth pattern is obtained upon intravenous cellular transfer of the leukaemic cells to other BN rats. Cytologically and cytochemically the disorder resembles human acute promyelocytic leukaemia and severe suppression of normal haematopoiesis occurs during the disease. In contrast to other leukaemia models the growth fraction is relatively low: 0.60 to 0.40 in the terminal phase of the disease. An i.v. inoculum of $10^7$ BNML cells kills the rat in 19–24 days. In this model a linear relationship is observed for the number of inoculated cells from $10^1$–$10^7$ cells and survival time (Colly *et al.*, 1984*b*).

### *Survival study*

Chemotherapy was initiated 14 days after i.v. inoculation of $10^7$ leukaemic cells into the BN rats. Twelve groups of rats were treated with three i.v. injections (q 12 h) of ara-C (50, 75, 100, 150, 200, 500 or 1,000 mg kg$^{-1}$) or aza-dC (5, 25, 50, 100 or 250 mg kg$^{-1}$), respectively. All dead animals were subjected to autopsy in order to judge the cause of death.

### *Cellular kinetics study*

One i.v. injection of ara-C or aza-dC, 200 mg kg$^{-1}$ or 100 mg kg$^{-1}$ respectively, was administered to BNML rats 15 days after inoculation with $10^7$ leukaemic cells. At the indicated times after the ara-C or aza-dC injection, two rats were sacrificed and bone marrow cells collected and treated separately. A fraction of these cells was prepared for cell cycle phase analysis by means of flow cytometry and DNA synthesis studies by means of $^3$HTdR uptake determination.

For flow cytometric analysis the nuclei of the bone marrow cells ($10^6$ cells ml$^{-1}$) were stained with the fluorescent dye propidium iodide. The stained cells were subjected to flow cytometry (FACS IV cell sorter, Becton and

Correspondence: D.J. Richel.
Received 27 March 1988; and in revised form, 25 July 1988.

Dickinson, Sunnyvale, Ca). The DNA histograms were analyzed with a Minc II (Digital Maynard, Ma) (Dean *et al.*, 1982). For $^{3}$HTdR uptake determination the bone marrow cells were resuspended in RPMI 1640 containing 5% foetal calf serum (Gibco) in a concentration of $2 \times 10^{5}$ cells ml$^{-1}$. These cell suspensions were incubated with 1 $\mu$Ci [$^{3}$H] methyl thymidine (20 Ci mmol$^{-1}$) for 1 h at 37°C in 5% $CO_2$. The cells were trapped on GF/C glass fibre filters (2.4 cm diam.) and washed with sterile 0.9% NaCl, cold 5% trichloroacetic acid and 96% ethanol. The disc was dried and placed in scintillation fluid to determine the amount of radioactivity incorporated into DNA.

*Colony forming unit-spleen assay (CFU-S)*

This method of quantifying the number of pluripotent haematopoietic stem cells (HSC) was first described by Till and McCulloch (1961) for mouse bone marrow cells, and later on modified for the rat (Van Bekkum, 1977). CFU-S were determined by pooling femoral bone marrow cells drawn from 3 normal rats at different time intervals after one i.v. injection of aza-dC or at 4 h after different doses of aza-dC. A fraction of these cells was injected into $F_1$ hybrids of C57 BL/$L_1$ Rij $\times$ $C_3H/L_w$ Rij mice, which had undergone 9.25 Gy total body irradiation.

Nine days after injection of the rat bone marrow cells, the mice were sacrificed and the spleens were harvested and fixed in Tellyesniczky's solution (ethanol 70%, formaldehyde 36%, acetic acid 100%, 20:1:1); the macroscopically visible colonies could easily be counted. A linear relationship was found between the number of cells injected and the number of spleen colonies formed (Van Bekkum, 1977).

### Results

*Effect of aza-dC and ara-C on survival of leukaemic rats*

Figure 1 shows the dose-response relationship, as indicated by the increase in median survival time (MST), for both drugs for BNML rats and non-treated leukaemic control animals. The increase in MST can be considered as a parameter of the antileukaemic effect. All animals died of progressive leukaemia, except 2 rats in the ara-C group, who died within 12 h after the first ara-C injection. These animals are presumed to be cases of toxic death and are therefore excluded from the data in Figure 1. For aza-dC, MST increased in a dose-dependent fashion for dosages up to 50 mg kg$^{-1}$; higher dosages did not produce a significant improvement in MST. The dose response relationship for low dose ara-C did not become clear in this study; at dosages exceeding 200 mg kg$^{-1}$ a slight increase in MST was observed. The MST curve for the aza-dC group is higher than that found for the ara-C group. At the highest dosages tested the increase in MST for ara-C was +14 days and for aza-dC +24 days ($P<0.001$ *t*-test).

**Figure 1** Dose-response relationship between increasing dosages of aza-dC or ara-C (each dose is given 3 times q 12h i.v.) and survival time after inoculation of $10^{7}$ leukaemic cells.

*Cellular kinetics of aza-dC and ara-C*

Figure 2 shows the effects of an i.v. injection of ara-C or aza-dC on DNA synthesis in leukaemic bone marrow cells. *In vitro* $^{3}$H-thymidine incorporation into bone marrow cells dropped sharply after an ara-C injection to ~50% of the initial value, followed by a sudden increase to ~3× the initial value 16 h after the injection and normalization at the end of the study.

In contrast to these observations, injection of aza-dC caused no changes in DNA synthesis; the results were comparable to those found for the control group.

The effects of ara-C and aza-dC on the percentage cells in S phase are shown in Figure 3. In this experiment the values for the percentage cells in S phase after ara-C are similar to those observed in previous studies (Colly *et al.*, 1984*a*) and show the pattern of synchronization of leukaemic cells, which is reflected in the accumulation of 50% cells in S phase. As in the $^{3}$HTdR uptake study no changes in the percentage cells in S phase occurred during the first 24 h after the aza-dC injection.

*Effect of ara-C and aza-dC on CFU-S*

Figure 4 shows the toxic effects of various dosages of ara-C and aza-dC on haematopoietic stem cells, as indicated by a decrease in the number of rat bone marrow colonies growing in the spleens of irradiated mice.

Even at the lowest dose of aza-dC (5 mg kg$^{-1}$) tested, the number of colonies dropped to ~60% of the initial value, a

**Figure 2** $^{3}$HTdR uptake studies of leukaemic bone marrow cells after injection of aza-dC (100 mg kg$^{-1}$ rapid i.v. ●——●), ara-C (200 mg kg$^{-1}$ rapid i.v. ▲——▲) or no drug (leukaemic controls □——□) into BNML rats. At the indicated hours after the injection 2 rats in each group were sacrificed and the leukaemic bone marrow cells were incubated separately with $^{3}$H thymidine.

**Figure 3** Comparison of percentage cells in S phase after injecting BNML rats with ara-C (200 mg kg$^{-1}$): ▲——▲ or aza-dC (100 mg kg$^{-1}$): ●——●. The leukaemic cells were obtained from the animals described in **Figure 2**.

plateau that persisted with increasing dosages. A similar pattern has been described for ara-C, although the plateau stabilized at ±75% of the initial value (Colly & Van Bekkum, 1982). This difference between aza-dC and ara-C, however, was not significant ($P = 0.05$, Mann–Whitney). The duration of suppression of the number of haematopoietic stem cells (CFU-S) produced by an injection of aza-dC ($5 mg kg^{-1}$) is shown in Figure 5. The plateau of 60% was still evident 12 h after injection.

### Discussion

In previous studies the effect of aza-dC on acute leukaemia in mice and *in vitro* was investigated. An important question about this deoxycytidine analogue is whether or not this agent has advantages over ara-C. Since experience with the Brown Norway rat leukaemia model indicated that ara-C is an effective drug with moderate toxic side effects, this animal model is very suitable for such a study; moreover it simulates in a more precise way the characteristics of human leukaemia (Colly *et al.*, 1984*b*, 1986).

**Figure 4** Dose-response relationship between increasing aza-dC doses and the number of CFU-S per $10^5$ nucleated bone marrow cells. Different symbols are the mean for different experiments and represent mean ± s.e. Inlay: comparable study for ara-C (Colly and Van Bekkum, 1982).

**Figure 5** Changes in the number of CFU-S per $10^5$ nucleated bone marrow cells at different time intervals after one aza-dC injection of $5 mg kg^{-1}$. Different symbols are the mean for different experiments and represent mean ± s.e.

Ara-C, the most active antileukaemic drug against human leukaemia, has been shown to increase the survival time for leukaemic Brown Norway rats in a weak dose-related fashion. A 5-fold increase in the dosage of $200 mg kg^{-1}$ resulted in only a slight prolongation of survival time in this model. Since $200 mg kg^{-1}$ body weight for a rat equals $1 g m^{-2}$ for man, this observation might indicate that the use of higher dosages of ara-C does not contribute to its antileukaemic effectiveness in man. For dosages up to $50 mg kg^{-1}$ aza-dC, survival time increases in a dose-related fashion; however, no further increase in MST was observed at higher dosages. The MST at this plateau level is however much longer than the MST found for rats treated with high-dose ara-C. Since in this model a linear relationship exists between the number of cells transplanted and survival of the recipients (Colly *et al.*, 1984*b*), the difference in the MST induced by each agent can be translated into the leukaemic cell kill. The increase in survival of 8–10 days induced by aza-dC means that 3 i.v. injections of the latter agent kills two logs more cells than 3 injections of ara-C (Colly *et al.*, 1984*b*).

In addition to its antileukaemic effect, the unique effects of ara-C on cell cycle kinetics (such as the $G_1$/S blockade and cell recruitment and synchronization) have been studied in the rat model (Colly *et al.*, 1984*a*). In this model manipulation of these phenomena led to optimal treatment programs. However, clinical application of this knowledge has been hampered by the heterogeneity among human leukaemias (Colly *et al.*, submitted). A comparison of the effects on the cell cycles of ara-C and aza-dC, as illustrated in Figures 3 and 4, reveals that the differences are striking. No alterations in cell cycle progression could be observed in the first 24 h after an injection of aza-dC. This is compatible with our observation in leukaemic cell lines, that growth inhibition and cytotoxic effects became evident ~2 cell cycles after incubation with aza-dC. Ultimately aza-dC induces cytotoxicity, as has been proven with colony formation assays (Momparler & Goodman, 1977).

It has been postulated that ara-C inhibits its own cell toxic actions by the $G_1$/S blockade: since the cells accumulate at the $G_1$/S boundary, they do not enter S phase and are thus protected against cytotoxicity. Delay of cell cycle progression at $G_1$/S could not be demonstrated for aza-dC, which might explain the greater antileukaemic activity observed.

Extreme haematological toxicity can limit the effectiveness of a cytostatic drug. Stem cell toxicity in man cannot be studied easily, but in the rat model it can be assessed by means of the CFU-S assay. Our study of ara-C and aza-dC showed that, in contrast to the dose-related antileukaemic activity, the maximum number of haematopoietic stem cells is already killed at relatively low dosages. Although there is a small difference between the two drugs in this respect, it is not significant. The latter observation combined with more effective antitumour effect of aza-dC implicates that aza-dC is a more effective antileukaemic agent than ara-C.

The difference between stem cell toxicity and anti-leukaemic effect favours treatment with high dosages of these agents, because the dose which causes maximal leukaemic cell kill is not more toxic for stem cells.

During the first 12 h after low dose aza-dC treatment ($5 mg kg^{-1}$) no recovery of initial stem cell values is observed. Longer follow up studies are required to study recovery from the toxic effect of aza-dC on stem cells. In summary it can be concluded that because of its increased antileukaemic activity, while the haematologic toxicity is comparable to ara-C, aza-dC might be very interesting clinically. In addition scientifically the many differences in biochemical properties with respect to ara-C also make it a fascinating agent.

### References

BEKKUM, D.W. VAN (1977). The appearance of the multipotential stem-cell (CFU-S). In *Experimental Haematology Today*, Baum, S.J. & Ledney, G.D. (eds) p. 3. Springer-Verlag: Berlin, New York.

BEKKUM, D.W. VAN & HAGENBEEK, A. (1977). Relevance of the BN leukaemia as a model for human acute myeloid leukaemia. *Blood Cells*, **3**, 565.

CHABOT, G.G. & MOMPARLER, R.L. (1986). Effects of 5 aza-2-deoxycytidine on survival and cell cycle progression of L1210 leukaemic cells. *Leukaemia Res.*, **10**, 533.

CLEUSAT, F. & CHRISTMAN, J.K. (1982). Inhibition of DNA methyltransferase and induction of Friend erythroleukaemia cell differentiation by 5-azacytidine and 5-aza-2-deoxycytidine. *J. Biol. Chem.*, **257**, 2041.

COLLY, L.P. & BEKKUM, D.W. VAN (1982). A recommendation for high-dose ara-C interval treatment based on studies in a slow growing leukaemia model (BNML). *Med. and Pediatric Oncol. Suppl.* **1**: 209.

COLLY, L.P., BEKKUM, D.W. VAN & HAGENBEEK, A. (1984*a*). Cell kinetic studies after high-dose ara-C and adriamycin treatment in a slowly growing rat leukaemia model (BNML) for human acute myelocytic leukaemia. *Leukaemia Res.*, **8**, 945.

COLLY, L.P., BEKKUM, D.W. VAN & HAGENBEEK, A. (1984*b*). Enhanced tumor load reduction after chemotherapy induced recruitment and synchronization in a slowly growing rat leukaemia model (BNML) for human acute myelocytic leukaemia. *Leukaemia Res.* **8**, 953.

COLLY, L.P., PETERS, W.G. & WILLEMZE, R. (1986). Effect of the interval between high dose 1-β-Arabinofuranosylcytosine injections on leukemic cell load, intestinal toxicity and normal hematopoietic stem cells in a rat model for acute myelogenous leukemia. *Cancer Res.*, **46**, 3825.

COLLY, L.P., PETERS, W.G. & WILLEMZE, R. (1988). Synchronization of leukaemic cells after high-dose cytosine arabinoside in patient with acute myelogenous leukaemia. Submitted.

DEAN, P.N., GRAY, J.W. & DOLBEAR, F.A. (1982). The analysis and interpretation of DNA distributions measured by flow cytometry. *Cytometry*, **3**, 188.

JONES, P.A. & TAYLOR, S.M. (1980). Cellular differentiation, cytidine analogs, and DNA methylation. *Cell.*, **20**, 85.

MAJOR, P., EGON, E.M., BEADSLEY, G., MINDEN, M. & KUFE, D.W. (1985). Lethality of human myeloblasts correlates with the incorporation of ara-C into DNA. *Proc. Natl Acad. Sci. USA*, **78**, 3235.

MOMPARLER, R.L. & GOODMAN, J. (1977). *In vitro* cytotoxic and biochemical effects of 5-aza-2-deoxycytidine. *Cancer Res.*, **37**, 1636.

MOMPARLER, R.L. & GONZALES, F.A. (1978). Effect of intravenous infusion of 5-aza-2-deoxycytidine on survival of mice with L1210 leukaemia. *Cancer Res.*, **38**, 2673.

MOMPARLER, R.L., RIVARD, G.E. & GYGER, M. (1985*a*). Clinical trial on 5-aza-2-deoxycytidine in patients with acute leukaemia. *Pharm. Ther.*, **30**, 277.

MOMPARLER, R.L., MOMPARLER, L.F. & SAMSON, J. (1985*b*). Comparison of the antileukaemic activity of 5-aza-2-deoxycytidine. 1-β-D-arabinofuranosylcytosine and 5-azacytidine against L1210 leukaemia. *Leukaemia Res.*, **8**, 1043.

TILL, J.E. & McCULLOCH, E.A. (1961). A direct measurement of the radiation sensitivity of normal mouse bone marrow cells. *Radiat. Res.*, **14**, 213.

VESELY, J. & CZHAK, A. (1977). Incorporation of a potent anti-leukaemic agent, 5-aza-2-deoxycytidine, into DNA of leukaemic cells from leukaemic mice. *Cancer Res.*, **37**, 3684.

WILSON, V.L., JONES, P.A. & MOMPARLER, R.L. (1983). Inhibition of DNA methylation in L1210 leukaemic cells by 5-aza-2-deoxycytidine as a possible mechanism of chemotherapeutic action. *Cancer Res.*, **43**, 3493.